# The anatomy of choice: active inference and agency

**DOI:** 10.3389/fnhum.2013.00598

**Published:** 2013-09-25

**Authors:** Karl Friston, Philipp Schwartenbeck, Thomas FitzGerald, Michael Moutoussis, Timothy Behrens, Raymond J. Dolan

**Affiliations:** ^1^The Wellcome Trust Centre for Neuroimaging, Institute of Neurology, University College LondonLondon, UK; ^2^Centre for Functional MRI of the Brain, The John Radcliffe HospitalOxford, UK

**Keywords:** active inference, agency, Bayesian, bounded rationality, embodied cognition, free energy, inference, utility theory

## Abstract

This paper considers agency in the setting of embodied or active inference. In brief, we associate a sense of agency with prior beliefs about action and ask what sorts of beliefs underlie optimal behavior. In particular, we consider prior beliefs that action minimizes the Kullback–Leibler (KL) divergence between desired states and attainable states in the future. This allows one to formulate *bounded* rationality as approximate Bayesian inference that optimizes a free energy *bound* on model evidence. We show that constructs like expected utility, exploration bonuses, softmax choice rules and optimism bias emerge as natural consequences of this formulation. Previous accounts of active inference have focused on *predictive coding* and Bayesian filtering schemes for minimizing free energy. Here, we consider *variational Bayes* as an alternative scheme that provides formal constraints on the computational anatomy of inference and action—constraints that are remarkably consistent with neuroanatomy. Furthermore, this scheme contextualizes optimal decision theory and economic (utilitarian) formulations as pure inference problems. For example, expected utility theory emerges as a special case of free energy minimization, where the *sensitivity* or inverse temperature (of softmax functions and quantal response equilibria) has a unique and Bayes-optimal solution—that minimizes free energy. This sensitivity corresponds to the *precision* of beliefs about behavior, such that attainable goals are afforded a higher precision or confidence. In turn, this means that optimal behavior entails a representation of confidence about outcomes that are under an agent's control.

## Introduction

This paper addresses the nature of probabilistic beliefs about control that constitute a sense of agency. By separating beliefs about control from action *per se*, one can formulate behavior as a pure inference problem. This allows one to describe goal-directed behavior and decision-making in terms of prior beliefs about how one should behave. It is these beliefs about controlled behavior that we associate with a representation or sense of agency. Here, we take a somewhat formal approach and illustrate the ideas using game theory and Markov decision processes. Our aim is to understand behavior in terms of approximate Bayesian inference and ask whether standard variational schemes can shed light on the functional anatomy of decision-making in the brain.

Our wider aim is to place heuristics in decision theory (in psychology) and expected utility theory (in economics) within the setting of embodied cognition or inference. In brief, we treat the problem of selecting a sequence of behaviors—to optimize some outcome—as a pure inference problem. We assume that policies are selected under the prior belief^1^ they minimize the divergence (relative entropy) between a probability distribution over states that can be reached and states agents believe they should occupy—states or goals that agents believe, *a priori*, have high utility. By formulating the problem in this way, three important aspects of optimal decision-making emerge:

- First, because relative entropy can always be decomposed into entropy and expected utility, the ensuing policies maximize expected utility and the entropy over final states. Entropy is a measure of average uncertainty (e.g., the entropy of a coin toss is much greater than the entropy of an unsurprising outcome, like the sun rising tomorrow). This decomposition is closely related to the distinction between extrinsic and intrinsic reward in embodied cognition and artificial intelligence. In this setting, utility or *extrinsic reward* is supplemented with *intrinsic reward* to ensure some efficient information gain, exploratory behavior or control over outcomes. Important examples here include artificial curiosity (Schmidhuber, 1991), empowerment (Klyubin et al., 2005), information to go (Tishby and Polani, 2011) and computational complexity (Ortega and Braun, 2011, 2013). Indeed, the causal generation of entropic forces in nonequilibrium systems has been proposed recently as a general mechanism for adaptive behavior (Wissner-Gross and Freer, 2013). In the present context, an intrinsically rewarding policy maximizes the opportunity to explore (or the entropy of) future states.
- Second, because policies are inferred, they are associated with a confidence or precision that is itself optimized. This furnishes a unique and Bayes-optimal sensitivity or inverse temperature—of the sort associated with softmax choice rules and quantal response equilibria (McKelvey and Palfrey, 1995).
- Third, because policy optimization is absorbed into the more general problem of inferring hidden states of the world, inferences about policies depend upon inferences about hidden states and *vice versa*. This means that beliefs about hidden states depend upon the confidence in policies—leading to an optimism bias (Sharot et al., 2012), in which inferences about ambiguous states are biased toward those that support an optimal policy.

In what follows, we motivate the premises that underlie this formulation and unpack its implications using formal arguments and simulations. The basic idea is that behavior can be cast as inference: in other words, action, and perception are integral parts of the same inferential process and one only makes sense in light of the other. It is fairly straightforward to show that self-organizing systems are necessarily inferential in nature (Friston, 2012). This notion dates back to the writings of Helmholtz and Ashby, who emphasized modeling and inference as necessary attributes of systems—like ourselves—that endure in a changing world (Helmholtz, 1866/1962; Ashby, 1947; Conant and Ashby, 1970). This idea has been formalized recently as minimizing a variational free energy bound on Bayesian model evidence—to provide a seamless link between occupying a limited number of attracting states and Bayesian inference about the causes of sensory input (Dayan et al., 1995; Friston, 2010). In the context of behavior, we suppose that inference underlies a sense of agency.

A corollary of this perspective is that agents must perform some form of active *Bayesian inference*. Bayesian inference can be approximate or exact, where exact inference is rendered tractable by making plausible assumptions about the approximate form of probabilistic representations—representations that are used to predict responses to changes in the sensorium. In general, exact inference is intractable and cannot be realized biophysically. This is because—for non-trivial models—the posterior distributions over unknown quantities do not have an analytic form. This means the challenge is to understand how agents perform approximate Bayesian inference. Conversely, in classical (normative) formulations, it is assumed that agents optimize some expected value or utility function of their states. The question then reduces to how the brain maximizes value (Camerer, 2003; Daw and Doya, 2006; Dayan and Daw, 2008).

Normative approaches assume that *perfectly rational* agents maximize value, without considering the cost of optimization (Von Neumann and Morgenstern, 1944). In contrast, *bounded rational* agents are subject to information processing costs and do not necessarily choose the most valuable option (Simon, 1956). Several attempts to formalize bounded rationality, in probabilistic terms, have focused on the Boltzmann distribution, where optimal behavior corresponds to picking states with a high value or low energy. In this setting, perfect rationality corresponds to choosing states from a low temperature distribution, whose probability mass is concentrated over the state with the highest value (Ortega and Braun, 2011). In particular, quantal response equilibrium (QRE) models of bounded rationality assume that choice probabilities are prescribed by a Boltzmann distribution and that rationality is determined by a *temperature* parameter (McKelvey and Palfrey, 1995; Haile et al., 2008). Boltzmann-like stochastic choice rules have a long history in the psychology and economics literature, particularly in the form of logit choice models (Luce, 1959; Fudenberg and Kreps, 1993). These choice rules are known as *softmax rules* and are used to describe stochastic sampling of actions, especially in the context of the exploration-exploitation dilemma (Sutton and Barto, 1998; Cohen et al., 2007). In this setting, the temperature parameter models the *sensitivity* of stochastic choices to value. This paper suggests that sensitivity can itself be optimized and corresponds to the confidence or precision associated with beliefs about the consequences of choices.

So what does active inference bring to the table? In active inference, there is no value function: free energy is the only quantity that is optimized. This means that bounded rationality must emerge from free energy minimization and the value of a state (or action) is a consequence of behavior, as opposed to its cause. In other words, the consequences of minimizing free energy are that some states are occupied more frequently than others—and these states can be labeled as valuable. We will see later that the frequency with which states are visited depends on prior beliefs—suggesting an intimate relationship between value and prior beliefs. Crucially, in active inference, parameters like sensitivity or inverse temperature must themselves minimize free energy. This means that sensitivity ceases to be a free parameter that is adjusted to describe observed behavior and becomes diagnostic of the underlying (approximate) Bayesian inference (that can be disclosed by observed choices). We will see later that sensitivity corresponds to the *precision* of beliefs about future states and behaves in a way that is remarkably similar to the firing of dopaminergic cells in the brain. Furthermore, QRE, logit choice models and softmax rules can be derived as formal consequences of free energy minimization, using variational Bayes.

Variational Bayes or ensemble learning is a ubiquitous scheme for approximate Bayesian inference (Beal, 2003). Variational Bayes rests on a partition or separation of probabilistic representations (approximate posterior probability distributions) that renders Bayesian inference tractable. A simple example would be estimating the mean and precision (inverse variance) of some data, under the assumption that uncertainty about the mean does not depend upon uncertainty about the variance and *vice versa*. This simple assumption enables a straightforward computation of descriptive statistics that would otherwise be extremely difficult: see (MacKay, 2003, p. 422) for details. In biological terms, a partition into conditionally independent representations is nothing more or less than functional segregation in the brain—in which specialized neuronal systems can be regarded as performing variational Bayesian updates by passing messages to each other. These messages ensure that posterior beliefs about states of (and actions on) the world are internally consistent. We will try to relate variational Bayes to the functional anatomy of inference and action selection in the brain. This provides a functional account of both neuronal representations and functional integration (message passing) among different systems.

Previous accounts of free energy minimization in the brain have focused on continuous time formulations and predictive coding as a neurobiologically plausible variational scheme. In this paper, we take a slightly more abstract approach and consider discrete time representations using variational Bayes. This necessarily implies a loss of biological realism; however, it provides an explicit model of discrete behaviors or choices. In particular, the resulting scheme converges, almost exactly, on the free energy formulation of decision-making under informational costs proposed by (Braun et al., 2011; Ortega and Braun, 2011). These authors accommodate nearly all optimal control, expected utility and evidence accumulation schemes under a single utility-based free energy minimization framework. The free energy minimization considered in this paper can be regarded as a special case of their general formulation, where the utility function is the log-likelihood of outcomes and their causes, under a generative model. This is important, because it connects utility-based schemes to variational Bayes and, more generally, inferential schemes that may underwrite biological self-organization (Ashby, 1947; Friston, 2012).

Although variational Bayes relies upon discrete updates, variational updates still possess a dynamics that can be compared to neuronal responses, particularly dopaminergic responses. In a companion paper (Friston et al., under review), we focus on this, because understanding the computational role of dopamine is important for understanding the psychopathology and pathophysiology of conditions like Parkinson's disease, schizophrenia and autism. In this paper, we focus on the functional anatomy implied by variational message passing in the brain and try to relate this to behavior from a psychological and economic perspective.

This paper comprises six sections: The first introduces active inference and sets up the basic ideas and notation. The second describes a fairly generic model of control or agency, in which purposeful behavior rests on prior beliefs that agents will minimize the (relative) entropy of their final states. We will see that this leads naturally to expected utility theory and exploration bonuses. The third section considers the inversion of this generative model using variational Bayes, with a special focus on mean field assumptions and implicit message passing. The fourth section considers the implications for the functional anatomy of inference and decision-making; namely, reciprocal message passing between systems supporting perceptual inference, action selection and evaluating precision. This section shows how key aspects of classical theory emerge; such as the distinction between perceptual inference about states of the world and action selection, quantal response equilibria, sensitivity and softmax choice rules. The fifth section uses simulations of a particular game (a waiting game with time sensitive contingencies) to illustrate the basic phenomenology of decision-making under active inference. The final section considers the cognitive anatomy of decision-making in terms of temporal discounting and marginal utility.

## Active inference

In active inference, beliefs about (hidden or fictive) states of the world maximize model evidence or the marginal likelihood of observations. In contrast to classic formulations, active inference makes a distinction between *action* as a physical state of the real world and beliefs about (future) action that we will refer to as *control* states—it is these that constitute a sense of agency. This changes the problem fundamentally from selecting an optimal action (a real variable) to making optimal inferences about control (a random variable). In other words, under the assumption that action is sampled from posterior beliefs about control, we can treat decision-making and action selection as a pure inference problem that necessarily entails optimizing beliefs about behavior and its consequences. This optimization appeals to the principle of free energy minimization.

### The free-energy principle and active inference

The free-energy principle (Friston et al., 2006) tries to explain how agents restrict themselves to a small number of attracting states. This behavior is equivalent to minimizing the Shannon entropy of the distribution over the outcomes they experience. Under ergodic assumptions, this entropy is (almost surely) the long-term time average of self-information or *surprise* (Birkhoff, 1931). Negative surprise ln *P*(*õ*|*m*) is the log likelihood of outcomes *õ* = (*o*_0_,…, *o_t_*), marginalized over their causes—also known as the *Bayesian model evidence* of model *m*. It is therefore sufficient to minimize surprise—at each point in time—to minimize its time average or Shannon entropy.

However, to evaluate surprise it is necessary to marginalize over the hidden causes of outcomes. This is the difficult problem of exact Bayesian inference. This problem can be finessed by using a proxy for surprise that does not depend on knowing the causes of observations. The proxy is variational free energy that, by construction, is an upper bound on surprise (Feynman, 1972; Hinton and van Camp, 1993). This means that if agents minimize free energy they minimize surprise (approximately). Coincidentally, they maximize model evidence (approximately) and implicitly engage in approximate Bayesian inference (Dayan et al., 1995; Friston, 2010). Put simply, although agents can never know the causes of their observations, the causes can be inferred. Crucially, the free energy that underpins this inference needs a generative model of how observations were caused—a model that can itself be optimized with respect to free energy (cf. Bayesian model selection in statistics).

These arguments suggest that action must minimize variational free energy, because outcomes can only be changed by action. This is *active inference* (Friston et al., 2010), which extends the minimization of free energy implicit in approximate Bayesian inference to include action. This means that behavior minimizes surprise or maximizes model evidence; either exactly—to produce perfectly rational behavior, or approximately—to minimize a variational bound to produce bounded rational behavior. There is a fairly developed literature on variational free energy minimization and active inference; covering things from perceptual categorization of bird songs, through to action observation (Friston, 2010). Most of this work uses generative models based upon differential equations. In this paper, we consider generative models based upon Markovian processes and revisit some of the key results in the context of decision-making and uncertainty.

In what follows, we use the usual conventions of uppercase to denote matrices and lowercase for vectors. In addition, we use bold typeface to indicate true variables in the world and italic typeface for hidden or fictive variables. The sufficient statistics (event probabilities) of categorical distributions over discrete states {1, …, *J*} are denoted by *J* × 1 vectors $\overset{⌢}{s}$ ϵ [0, 1]. The ~ notation denotes collections of variables over time.

#### Definition

Active inference rests on the tuple (Ω, **S**, *A, P, Q, R, S, U*):

- A finite set of observations Ω
- A finite set of true states and actions **S** × *A*
- A finite set of fictive or hidden states *S* × *U*
- A *generative process* over observations, states and action
  $$R\left(\tilde{o},\tilde{s},a\right)=\text{Pr}\left(\{o_{0},\ldots ,o_{t}\}=\tilde{o},\{s_{0},\ldots ,s_{t}\}=\tilde{s},A=a\right)$$
- A *generative model* over observations and hidden states
  $$\begin{array}{l} P(\tilde{o},\tilde{s},\tilde{u}|m)=\mathrm{Pr}(\{o_{0},\ldots ,o_{t}\}=\tilde{o},\{s_{0},\ldots ,s_{t}\}=\text{​}\tilde{s},\\ \;\{u_{0},\ldots ,u_{T}\}=\tilde{u}) \end{array}$$
- An *approximate posterior probability* over hidden states with sufficient statistics μ ϵ **R**^*d*^ such that
  $$Q(\tilde{s},\tilde{u}|\mu )=\mathrm{Pr}(\{s_{0},\ldots ,s_{t}\}=\tilde{s},\{u_{0},\ldots ,u_{T}\}=\tilde{u}),$$

#### Remarks

Here, *m* denotes the form of a generative model or probability distribution entailed by an agent. For clarity, we will omit the conditioning on *m* unless necessary. In this setup, the *generative process* describes transitions among real states of the world that depend upon action and generate outcomes. The agent is equipped with a *generative model* of this process, where action is replaced by a subset of hidden states called control states *U*. Although we allow for any action (control) from any state, only a subset may be allowable from any given state. Finally, the sufficient statistics of the approximate posterior encode a probability distribution over hidden states *S* × *U* at times *t* ϵ {0, …, *T*}. In other words, the sufficient statistics—or parameters of the distribution—represent the probability of hidden states.

As it stands, the above definition does not describe a process. This is because the dependencies among real states and sufficient statistics are not specified. In other words, the agent's generative model of observations *P*(*õ*, $\tilde{s}$, *ũ*|*m*) and its approximate posterior distribution over their causes *Q*($\tilde{s}$, *ũ*|μ) does not refer to the process of eliciting outcomes through action *R*(*õ*, $\tilde{s}$, *a*). To couple the agent to its environment, we have to specify how its sufficient statistics depend upon observations and how its action depends upon sufficient statistics. In active inference, the sufficient statistics minimize free energy and the ensuing beliefs about control states prescribe action:

(1) $$\begin{array}{l} \;\mu_{t}={\text{argmin}}_{\mu }F\left(\tilde{o},\mu \right)\\ \text{Pr}\left(a_{t}=u_{t}\right)=Q\left(u_{t}|\mu_{t}\right) \end{array}$$

This is usually portrayed in terms of *perception* (inference about hidden states) and *action* (a choice model in which action is a function of inferred states). Usually, sufficient statistics are associated with the internal states of an agent (such as neuronal activity or connection strengths) and action is associated with the state of its effectors. In more general formulations, action would select outcomes with the lowest free energy (Friston et al., 2012a). However, for simplicity, we have assumed that actions are sampled from posterior beliefs about control states—noting that the actions which minimize free energy produce outcomes that are the most likely under posterior beliefs. In short, sufficient statistics and implicit posterior beliefs about the state of the world minimize free energy, while action is selected from posterior beliefs about control states. We will see later that these posterior beliefs depend crucially upon prior beliefs about states that will be occupied in the future.

**Figure 1:**
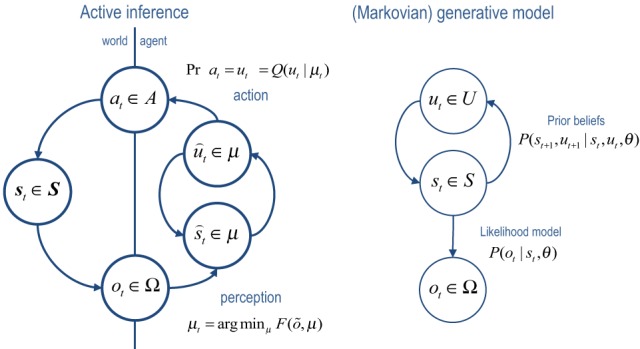
**Left panel:** this is a schematic of the dependencies among variables underlying active inference. Here, a generative process representing state transitions in the real world generates observations or outcomes that are used to update the internal states of an agent. These internal states encode the sufficient statistics of an approximate posterior distribution over variables defined by a generative model (right panel). Particular sufficient statistics, encoding beliefs about choices or control states are reflexively transcribed into action, which affects real state transitions—thereby closing the action–perception cycle. **Right panel**: notice that the generative model, which defines free energy has a much simpler form. It simply supposes that there are mutually dependent hidden and control states that conspire to produce observations.

Figure 1 provides a schematic of the resulting cycle of action and perception, where posterior expectations (sufficient statistics) minimize free energy and prescribe action (left panel). In this setting, free energy is defined in relation to the generative model (right panel). Notice that the generative model does not need to know about action: from its point of view, the world contains (fictive) control states that determine transitions among hidden states generating outcomes. In other words, optimizing posterior beliefs about control states produces action automatically but the agent does not know this—in the sense we are aware of the sensory consequences of our motor reflexes but not the reflexes *per se*.

One can express free energy in a number of ways:

(2) $$\begin{array}{l} F\left(\tilde{o},\mu \right)=E_{Q}\left[-\text{ln}P\left(\tilde{o},\tilde{s},\tilde{u}|m\right)\right]-H\left[Q\left(\tilde{s},\tilde{u}|\mu \right)\right]\\ \;=D_{KL}\left[Q\left(\tilde{s},\tilde{u}|\mu \right)\text{​}||\text{​}P\left(\tilde{s},\tilde{u}|\tilde{o}\right)\right]-\text{ln}P\left(\tilde{o}|m\right)\\ \;=D_{KL}\text{​}\left[Q\text{​}\left(\tilde{s},\tilde{u}|\mu \right)\text{​}||\text{​}P\text{​}\left(\tilde{s},\tilde{u}|m\right)\right]+E_{Q}\text{​}\left[-\ln P\left(\tilde{o}|\tilde{s},\tilde{u}\right)\text{​}\right] \end{array}$$

The first equality expresses free energy as a Gibbs energy (expected under the approximate posterior) minus the entropy of the approximate posterior. This speaks to why it is called a free energy. The second equality shows that free energy is an upper bound on surprise, because the first relative entropy or Kullback–Leibler (KL) divergence term is non-negative by Gibbs inequality (Beal, 2003). This means minimizing free energy corresponds to minimizing the divergence between the approximate and true posterior. This formalizes the notion of unconscious inference in perception (Helmholtz, 1866/1962; Dayan et al., 1995; Dayan and Hinton, 1997) and—under some simplifying assumptions—reduces to predictive coding (Rao and Ballard, 1999). The third equality shows that minimizing free energy is the same as maximizing the expected log likelihood of observations or *accuracy*, while minimizing the divergence between the approximate posterior and prior beliefs about hidden variables. This divergence is known as *model complexity* (Spiegelhalter et al., 2002; Penny et al., 2004), ensuring that inference is both accurate and parsimonious (cf. Occam's razor).

In summary, if agents resist a natural tendency to disorder (occupy a limited number of characteristic states), then they become implicit Bayesian modelers of their environment. This is consistent with the good regulator hypothesis (Conant and Ashby, 1970) and accounts of (unconscious) inference and perception in the brain (Helmholtz, 1866/1962; Gregory, 1968; Dayan et al., 1995). Crucially, this requires agents to entertain beliefs about the control of state transitions producing outcomes. This means we have moved beyond classic formulations—in which deterministic actions are selected—and have to consider posterior beliefs about putative choices. These beliefs determine the states that are eventually sampled. In the next section, we consider the optimization of posterior beliefs; both in terms of their content and the confidence or precision with which they are held.

## A generative model of agency

We have seen that a generative model is necessary to furnish a free energy bound on surprise or Bayesian model evidence. This model comprises prior beliefs that determine the states an agent or model will frequent. These beliefs specify the attracting states (goals) that action will seek out. In this section, we consider the form of these beliefs and how they can be understood in terms of expected utility.

### The generative model

**Figure 2:**
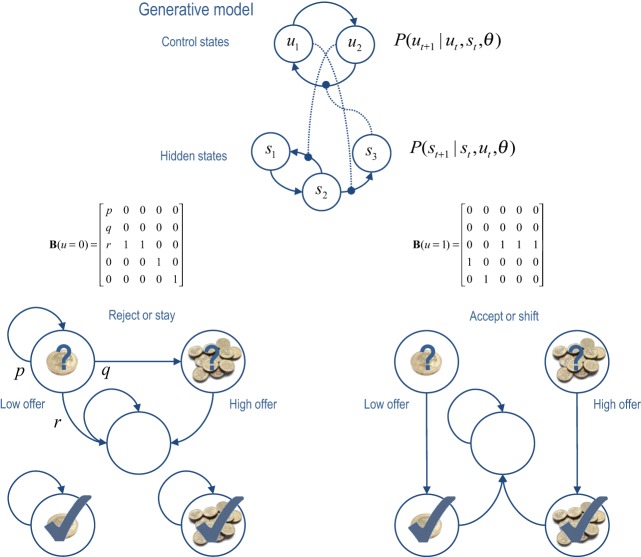
**Upper panel:** this is an example of a generative model, based on a hierarchical hidden Markov model. The key feature of this model is that there are two sets of states; hidden states and control states. The transitions among one set of states depend upon the state occupied in the other set. **Lower panels**: this provides an example of a particular generative model in which there are two control states; reject (stay) or accept (shift). The control state determines the transitions amongst the hidden states which, in this example, comprise a low offer (first state), a high offer (second state), a no-offer state (third state), and absorbing states that are entered whenever a low (fourth state) or high (fifth state) offer is accepted. The probability of moving from one state to another is one, unless specified by the values of the (control dependent) transition probabilities shown in the middle row. For example, the parameter *r* controls the rate of offer withdrawal (cf. a hazard rate). Note that absorbing states—that re-enter themselves with unit probability—render this Markovian process irreversible. We will use this example in simulations of choice behavior.

The Markovian models considered here rest on transitions among hidden states that are coupled to transitions among control states. This is illustrated in terms of a hidden Markov model or finite state (epsilon) machine (Ellison et al., 2011) in the upper panel of Figure 2. In the general forms of these models, control states modify the transition probabilities among hidden states, while hidden states modify the transitions among control states (as denoted by the connections with circles). This sort of model allows context-sensitive transitions among states generating outcomes—that themselves can induce changes in the control states providing the context. The lower panels of Figure 2 illustrate a particular example that we will use later—in which there are two states that control transitions among five hidden states (see figure legend for details).

The generative model used to model these (irreversible Markovian) processes can be expressed in terms of future control states *ũ* = (*u_t_*, …, *u_T_*) as follows:

(3) $$\begin{array}{l} \;P\text{​}\left(\tilde{o},\tilde{s},\tilde{u},\gamma |\tilde{a,m}\right)=P\left(\tilde{o}|\tilde{s}\right)P\left(\tilde{s},\tilde{u}|\gamma ,\tilde{a}\right)P\left(\gamma |m\right)\\ \;P\text{​}\left(o_{0},\ldots ,o_{t}|s_{0},\ldots ,s_{t}\right)=\prod_{i=0}^{t}P(o_{i}|s_{i})\\ P\text{​}\left(s_{0},\ldots ,s_{t},\tilde{u}|\gamma ,a_{0},\ldots ,a_{t-1}\right)=P(\tilde{u}|s_{t})P(s_{0}|m)\prod_{i=1}^{t}\\ \;P(s_{i}|s_{i-1},a_{i-1})\\ \;\ln P\text{​}\left(\tilde{u}|s_{t}\right)=-\gamma \cdot D_{KL}[P(s_{T}|s_{t},\tilde{u})||P(s_{T}|m)] \end{array}$$

#### Remarks

The first equality expresses the model in terms of the likelihood of observations given the hidden and control states (first term) and *empirical* prior beliefs (subsequent terms). Empirical priors are probability distributions over unknown variables that depend on other unknown variables—and are an inherent part of any hierarchical model. The likelihood (second equality) says that observations depend on, and only on, concurrent hidden states. The third equality expresses beliefs about state transitions that embody Markovian dependencies among successive hidden states. For simplicity, we have assumed that the agent knows its past actions by observing them.

The important part of this model lies in the last equality. This describes prior beliefs about control sequences or *policies* that determine which actions are selected. These beliefs take the form of a Boltzmann distribution, where the policy with the largest prior probability minimizes the relative entropy or divergence between the distribution over final states, given the current state and policy, and the marginal distribution over final states. This marginal distribution encodes goals in terms of (desired) states the agent believes it should visit from current state. Crucially, the precision of beliefs about policies is determined by a hidden variable γ ϵ ℝ^+^ that has to be inferred. In essence, this model represents past hidden states and future choices, under the belief that control from the current state will minimize the divergence between the distribution over final states and a prior distribution or goal.

### Prior beliefs, entropy and expected utility

Basing beliefs about future choices on relative entropy is formally related to optimization schemes based on KL control; particularly risk sensitive control; e.g., (van den Broek et al., 2010). This is also a cornerstone of utility-based free energy treatments of bounded rationality (Ortega and Braun, 2011). These schemes consider optimal agents to minimize the KL divergence between controlled and desired outcomes. All we have done here is to equip agents with a sense of agency or prior beliefs that they are KL optimal. These beliefs are then enacted through active inference. The advantage of this is that the precision of beliefs about control can now be optimized—because we have effectively cast the optimal control problem as an inference problem. These arguments may seem a bit abstract but, happily, concrete notions like exploration, exploitation and expected utility emerge as straightforward consequences:

The relative entropy or divergence can be thought of as a prediction error that is nuanced in an important way: it reports the mismatch—not between expected and observed outcomes—but between the final outcomes expected with and without considering the current state: in other words, the difference between what can be attained from the current state and the goals encoded by prior beliefs. Unlike classic reward prediction errors, this probabilistic prediction error is a difference between probability distributions over states. Mathematically, this divergence can be decomposed into two terms that have important implications for behavior. From Equation 3:

(4) $$\begin{array}{l} \ln P\left(\tilde{u}|s_{t}\right)=\gamma \cdot Q\\ \;Q\left(\tilde{u}|s_{t}\right)=-D_{KL}\left[P\left(s_{T}|s_{t},\tilde{u}\right)\text{​}||P\left(s_{T}|m\right)\right]\\ \;=\sum_{s_{T}}P\left(s_{T}|s_{t},\tilde{u}\right)\ln\frac{P\left(s_{T}|m\right)}{P\left(s_{T}|s_{t},\tilde{u}\right)}\\ \;=\underset{\text{exploration bonus}}{\underset{︸}{H\left[P\left(s_{T}|s_{t},\tilde{u}\right)\right]}}+\sum_{s_{T}}\underset{\text{expected utility}}{\underset{︸}{P\left(s_{T}|s_{t},\tilde{u}\right)c\left(s_{T}|m\right)}} \end{array}$$

This expresses the log likelihood of a policy as a precision weighted value *Q*(*ũ*|*s_t_*). This *value* is an attribute of policies available from the current state, where the value of a policy is the negative divergence between the states entailed by the policy and goal states. In other words, a valuable policy (or state) minimizes relative entropy. We use *Q*(*ũ*|*s_t_*) to emphasize the analogous role of action value in Q-learning (Watkins and Dayan, 1992). Equation 4 shows that value can be decomposed into terms. The first is the entropy (intrinsic reward) of the distribution over final states, given the current state and policy. The second is the expected *utility* of the final state, where utility (extrinsic reward) or negative cost is the log probability of the final state, under the prior goals *c*(*s_T_*|*m*) = ln *P*(*s_T_*|*m*).

These definitions help us connect to classic formulations and highlight an important difference between the value of choices and the utility of states. Utility is a fixed attribute of states that agents are attracted to. In contrast, the value of a policy is context sensitive and depends upon the current state. Because utility is defined in terms of a probability distribution—which sums to one—the utility (log probability) of any state is negative and can be at most zero (i.e., cost is non-negative). This setup highlights the relative nature of utility (Tobler et al., 2005; Jocham et al., 2012), because the value of a policy is determined by the difference among the utilities of outcomes.

### Exploration, exploitation and novelty

This decomposition of value means that agents (believe they) will maximize the entropy of their final states while maximizing expected utility. The relative contribution of entropy and expected utility depends upon the precision of prior beliefs about the final state or, equivalently, the relative utility of different states. If these beliefs are very precise (informative), they will dominate and the agent will (believe it will) maximize expected utility. Conversely, with imprecise (flat) prior beliefs that all final states are equally valuable, the agent will try to keep its options open and maximize the entropy over those states: in other words, it will explore according to the maximum entropy principle (Jaynes, 1957). This provides a simple account of *exploration-exploitation* that is consistent with expected utility theory. The entropy term implies that (beliefs about) choices are driven not just to maximize expected value but to explore all options in a way that confers an exploratory aspect on behavior. In the absence of (or change in) beliefs about ultimate states, there will be a bias toward visiting all (low cost) states with equal probability. Similarly, the *novelty bonus* (Kakade and Dayan, 2002) of a new state is, in this formulation, conferred by the opportunity to access states that were previously unavailable—thereby increasing the entropy over final states. As indicated in Equation (4), this means that the value of a choice comprises an exploration bonus and an expected utility, where the former depends upon the current state and the latter does not.

In summary, if agents occupy a limited set of attracting states, it follows that their generative models must be equipped with prior beliefs that controlled state transitions will minimize the divergence between a distribution over attainable states and a distribution that specifies states as attractive. These prior beliefs can be expressed in terms of relative entropy that defines the value of policies. This value has exactly the same form as the objective functions in KL control schemes that grandfather conventional utility-based schemes (Kappen et al., 2012; Ortega and Braun, 2011). The value of a policy can be decomposed into its expected utility and an exploration or novelty bonus that corresponds to the entropy over final states. In this setting, notions like value, expected utility and exploration bonus are consequences of the underlying imperative to minimize (relative) entropy, entailed by the priors of an agent's generative model.

The balance between exploration (entropy) and exploitation (expected value) is uniquely determined by the relative utility of future states and not by the temperature parameter—the precision or inverse temperature applies to both exploratory and utilitarian behavior (see Equation 4). In other words, explorative behavior is not just a random version of exploitative behavior but can itself be very precise, with a clearly defined objective (to maximize the entropy of final outcomes). In fact, precision plays a fundamental role in moderating an *optimism bias* when forming beliefs about hidden states of the world (Sharot et al., 2012). To see this clearly, we need to consider the nature of model inversion.

## Variational bayesian inversion

This section illustrates active inference using variational Bayesian inversion of the generative model above. To simplify notation, we will represent allowable policies with π ϵ {1, …, *K*}, were each policy prescribes a sequence of control states (*ũ*|π) = (*u_t_*, …, *u_T_*|π). The model considered in the remainder of this paper is parameterized as follows:

(5) $$\begin{array}{l} \;P\left(o_{t}=i|s_{t}=j,A\right)=A_{ij}\\ \;P\left(s_{t+1}=i|s_{t}=j,\pi ,B\right)=B{\left(u_{t}|\pi \right)}_{ij}\\ \ln P\left(\pi =i|s_{t}=j,\gamma ,Q\right)=Q_{ij}\cdot \gamma -\ln Z_{\pi }\\ \;P\left(s_{T}=i|c\right)=c_{i}\\ \;P\left(s_{0}=i|d\right)=d_{i}\\ \;P\left(\gamma |m\right)=\Gamma (\alpha ,\beta )\\ \;P\left(s_{T}=i|s_{t}=j,\pi ,c\right)=T{(\pi )}_{ij}\\ \;T(\pi )=B(u_{t}|\pi )B(u_{t+1}|\pi )\ldots B(u_{T}|\pi )\\ \;Q_{ij}=\ln c^{T}\cdot T{(\pi =i)}_{j}-\ln T{\left(\pi =i\right)}_{j}^{T}\cdot T{(\pi =i)}_{j}\\ \;\sum_{i}A_{ij}=1,\sum_{i}B{\left(u_{t}\right)}_{ij}=1,\sum_{i}c_{i}=1,\sum_{i}d_{i}=1 \end{array}$$

Categorical distributions over observations, given the hidden states, are parameterized by the matrix *A* that maps, probabilistically, from hidden states to outcomes. Similarly, the transition matrices *B*(*u_t_*) encode transition probabilities from one state to the next, under the current control state of a policy. The vectors **c** and **d** encode the prior distribution over the last and first states, respectively. The former parameters specify the priors on control, where utility is *c*(*s_T_*|*m*) = ln *P*(*s_T_*|*m*) = ln **c**. The prior over precision has a gamma distribution with shape and rate parameters (in this paper) α = 8 and β = 1.

The *K* × *J* matrix *Q* contains the values of allowable policies from current states, where the normalization constant *Z*_π_ ensures that the probabilities over policies sum to one. Finally, the matrices *T*(π) encode the probability of transition from the current state to a final state, under a particular policy. This is the composition of transition matrices from the present time until the end of the game. Transition probabilities to the final state determine the entropy and expected utility that comprise value (last equality). Here, *T*(π = *i*)_*j*_ is a column vector of probabilities over final states, under the *i*-th policy and *j-th* current state.

### Approximate bayesian inference

Having specified the exact form of the generative model, we now need to find the sufficient statistics of the approximate posterior density that minimizes free energy. This is equivalent to approximate Bayesian inference about hidden variables ψ =($\tilde{s}$, *ũ*, γ). Variational Bayes now provides a generic and relatively simple scheme for approximate Bayesian inference that finesses the combinatoric and analytic intractability of exact inference (Beal, 2003; Fox and Roberts, 2012).

The efficiency of variational Bayes rests on replacing posterior dependencies among hidden variables with dependencies among the sufficient statistics of marginal probabilities over subsets. This allows one to factorize the (approximate) posterior distribution into marginal distributions, which greatly reduces the size of the state space that has to be represented. This is because one does not have to represent the joint distribution over different subsets. To illustrate this, consider a distribution over all combinations of *J* hidden states and *K* control states at every point in time: *Q*($\tilde{s}$, *ũ*). The underlying state space *S*_1_ × *U*_1_ × … × *S_T_* × *U_T_* would require an untenable number (*J* × *K*)^*T*^ of sufficient statistics or probabilities—the example below would require (5 × 2)^16^ sufficient statistics, which is more than the number of synapses in the brain.

**Figure 3:**
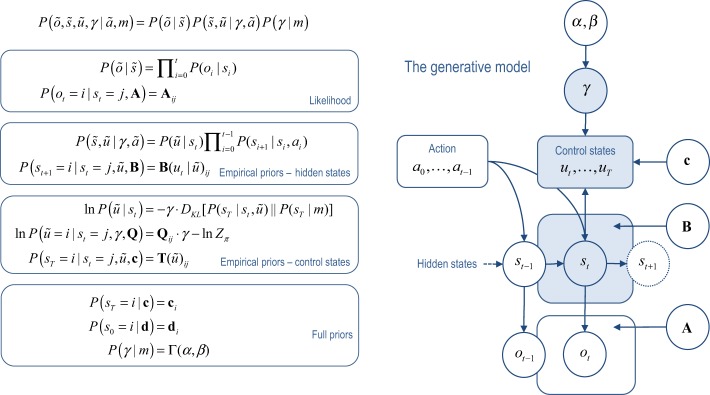
**This figure illustrates the temporal dependencies among hidden states and control states in the generative model considered in this paper**. This Bayesian graph illustrates the dependencies among successive hidden states and how they depend upon action in the past and control states in the future. Note that future control states depend upon the current state because it depends upon the relative entropy or divergence between distributions over the final state that are, and are not, conditioned on the current state. The resulting choices depend upon the precision of beliefs about control states, which, in turn depend upon the parameters of the model. Observed outcomes depend on, and only on, the hidden states at any given time.

However, if we exploit the Markovian dependencies among successive states, we can use a *mean field assumption* to reduce the number of sufficient statistics dramatically. The particular mean field assumption we will use is (see also Figure 3):

(6) $$\begin{array}{l} Q(\tilde{s},\tilde{u},\gamma |\mu )=Q(s_{0}|\text{​​}\overset{⌢}{s_{0}})\ldots Q(s_{t}|\text{​​}\overset{⌢}{s_{t}})Q(\tilde{u}|\text{​​}\overset{⌢}{\pi })Q(\gamma |\text{​​}\overset{⌢}{\beta })\\ \;Q(s_{t}=j|\text{​​}\overset{⌢}{s_{t}})=\overset{⌢}{s}\text{​​}{\text{​}}_{tj}\text{​}:\sum_{j}\overset{⌢}{s}\text{​}{\text{​}}_{tj}=1\\ \;Q(\tilde{u}=k|\text{​​}\overset{⌢}{\pi })={\overset{⌢}{\pi }}_{k}:\sum_{k}{\overset{⌢}{\pi }}_{k}=1\\ \;Q(\gamma |\text{​​}\overset{⌢}{\beta })=\Gamma (\alpha ,\text{​​}\overset{⌢}{\beta }) \end{array}$$

Here, we have assumed a factorization over (past) hidden states, (future) control states and precision. Furthermore, we have factorized successive states over time, which means we only have to represent the current state explicitly. These particular mean field assumptions are not approximations, because the true generative process is Markovian. Conversely, the factorization with respect to precision is an approximation, because the true posterior will show (mild) conditional dependencies between precision and hidden states.

The marginal over control states has not been factorized because the final outcome depends, in general, on the particular history of choices. In other words, generally speaking, any outcome depends upon the sequence of choices in the past. However, there are potentially a vast number of control sequences or policies that could require an enormous number of sufficient statistics. This problem can be finessed by only considering allowable or *a priori* plausible policies. In the example below, there is no point in accepting an offer more than once. Therefore, we only need to consider policies in which an offer is accepted once during the game. There is nothing lawful about this restriction; however, it is particularly appropriate for irreversible Markovian processes that have absorbing states (that render further action pointless). These processes are ubiquitous in game theory where, having made a choice, there is no going back. This allows one to reduce the number of sufficient statistics for policies from *K*^*T*^ to (*K* − 1) × *T* by only allowing policies in which a choice *u*_τ_ > 1 is made at *t* = τ and *u*_*t*_ = 1 otherwise.

The details of the mean field assumption above are not terribly important. The main point here is that the formalism of variational Bayes allows one to specify constraints on the form of the approximate posterior that makes prior assumptions or beliefs about allowable choices explicit. For example, in (Friston et al., 2012a) we used a mean field assumption where every choice could be made at every time point. Equation (6) assumes the approximate marginal over precision is, like its conjugate prior, a gamma distribution; where the shape parameter is the same as the prior α = 8 and the rate parameter is optimized. This rate parameter corresponds to temperature in classic formulations. Crucially, it is no longer a free parameter but a sufficient statistic of the unknown precision of beliefs about policies.

### Variational updates

Variational Bayes optimizes the sufficient statistics μ ϵ ℝ^+^ with a series of variational updates. It is straightforward to show (Beal, 2003) that the marginal distributions *Q*(ψ_*i*_ |μ_*i*_) that minimize free energy can be expressed in terms of the *variational energies V*(ψ_*i*_) of each subset:

(7) $$\begin{array}{l} \ln Q\left(\psi_{i}|\mu_{i}\right)=V\left(\psi_{i}\right)+\ln Z_{i}\Rightarrow \frac{\partial F\left(\tilde{o},\mu \right)}{\partial \mu_{i}}=0\\ \;V\left(\psi_{i}\right)=E_{Q\left(\psi_{\backslash i}\right)}\left[\ln P(\tilde{o},\psi |m)\right]\\ \;\psi =(s_{0},\ldots ,s_{t},\tilde{u},\gamma )\\ \;\mu =(\overset{⌢}{s_{0}},\ldots ,\overset{⌢}{s_{t}},\overset{⌢}{\pi },\;\overset{⌢}{\beta }) \end{array}$$

The variational energies are just the (negative) Gibbs energies in Equation (2), expected under the Markov blanket *Q*(ψ_\*i*_) of each subset. Loosely speaking, the Markov blanket contains all subsets, apart from the subset in question. The important thing about this result is that it expresses the optimal sufficient statistics of one subset in terms of the others. This allows one to iteratively re-evaluate each subset, given the others, until convergence. This is, in essence, variational Bayes. Given the generative model in Equation (5) and the mean field assumption in Equation (6), Equation (7) furnishes the following remarkably simple updates (starting from prior beliefs):

(8) $$\begin{array}{l} \;\overset{⌢}{s_{t}}=\sigma \left(\ln A^{T}\cdot \overset{⌢}{o_{t}}+\ln B\left(a_{t-1}\right)\cdot \overset{⌢}{s_{t-1}}+\overset{⌢}{\gamma }\cdot Q^{T}\cdot \overset{⌢}{\pi }\right)\\ \;\overset{⌢}{\pi }=\sigma \left(\overset{⌢}{\gamma }\cdot Q\cdot \overset{⌢}{s_{t}}\right)\\ \;\overset{⌢}{\;\beta }=\beta -{\overset{⌢}{\pi }}^{T}\cdot Q\cdot \overset{⌢}{s_{t}}\\ \;\overset{⌢}{\gamma }=\frac{\alpha }{\overset{⌢}{\beta }}\\ \sigma \left(V\right)=\frac{\text{exp​}\left(V\right)}{\sum_{i,j}\text{exp​}\left(V_{ij}\right)} \end{array}$$

These expressions follow in a straightforward way from the variational energies in Equation (7): see the **Appendix** and (Beal, 2003) for details. These updates assume the parameters of the model are known. If they are not, then it is relatively straightforward to extend the variational Bayesian scheme above to include variational updates for *learning* unknown parameters, as described in Chapter 3 of (Beal, 2003). The only special consideration is the use of conjugate (Dirichlet) priors over the parameters.

In summary, variational Bayes involves iterating updates to find the sufficient statistics that minimize free energy and, implicitly, provide Bayesian estimates of the hidden variables. This means the sufficient statistics change over two timescales—a fast timescale that updates posterior beliefs given the current observations—and a slow timescale that updates posterior beliefs as new observations become available and action is taken. We have previously speculated (Friston et al., 2012a) that this separation of temporal dynamics may be related to nested electrophysiological oscillations, such as phase coupling between gamma and theta oscillations in prefrontal–hippocampal interactions (Canolty et al., 2006). This speaks to biological implementations of variational Bayes, which we now consider in terms of neuronal and cognitive processing.

## The functional anatomy of decision-making

**Figure 4:**
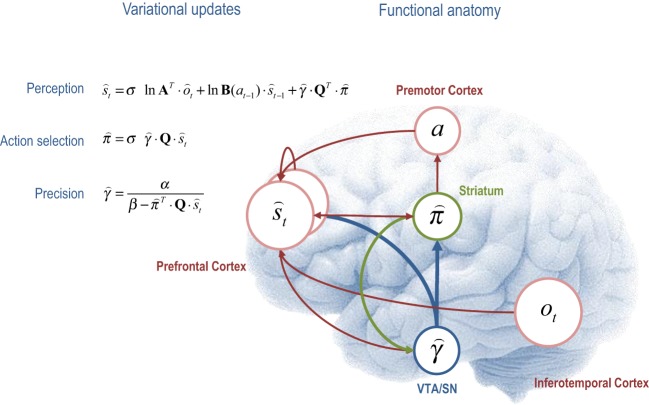
**This figure illustrates the cognitive and functional anatomy implied by the variational scheme—or more precisely, the mean field assumption implicit in variational updates**. Here, we have associated the variational updates of expected states with perception, of future control states (policies) within action selection and, finally, expected precision with evaluation. The forms of these updates suggest the sufficient statistics from each subset are passed among each other until convergence to an internally consistent (Bayes optimal) solution. In terms of neuronal implementation, this might be likened to the exchange of neuronal signals via extrinsic connections among functionally specialized brain systems. In this (purely iconic) schematic, we have associated perception (inference about the current state of the world) with the prefrontal cortex, while assigning action selection to the basal ganglia. Crucially, precision has been associated with dopaminergic projections from the ventral tegmental area and substantia nigra that, necessarily, project to both cortical (perceptual) and subcortical (action selection) systems. See main text for a full description of the equations.

The variational scheme above has a computational form that resembles many aspects of neuronal processing in the brain. If we assume that neuronal activity encodes sufficient statistics, then the variational update scheme could provide a metaphor for *functional segregation*—the segregation of representations corresponding to the mean field assumption, and *functional integration*—the recursive (reciprocal) exchange of sufficient statistics during approximate Bayesian inference. In terms of the updates themselves, the expectations of hidden states and policies are softmax functions of mixtures of the other expectations. This is remarkable because these updates are derived from basic variational principles and yet they have exactly the form of neural networks that use integrate and fire neurons—and are not dissimilar to real neurons with sigmoid activation functions. Furthermore, the softmax functions are of linear mixtures of sufficient statistics (neuronal activity) with one key exception; namely, the modulation by precision when updating beliefs about the current state of the world and selecting the next action. It is tempting to equate this modulation with the neuromodulation by ascending neurotransmitter systems such as dopamine that send projections to (prefrontal) systems involved in working memory (Goldman-Rakic, 1997; Moran et al., 2011) and striatal systems involved in action selection (O'Doherty et al., 2004; Surmeier et al., 2009). We now consider each of the variational updates from a cognitive and neuroanatomical perspective (see Figure 4 for a summary):

### Perception

The first variational step updates beliefs about the current state of the world using observed outcomes and representations of the preceding state. This confers a temporal contiguity on inference, where empirical prior beliefs about the current state conspire with sensory evidence to produce posterior beliefs. However, there is a third term that corresponds to expected value of each state, averaged over policies. This term can be thought of as an optimism bias in the sense that, when precision is high, perception is biased toward the state that has the greatest potential to realize the agent's goal. We can now see why precision moderates this bias, much like dopamine (Sharot et al., 2012). Figure 4 ascribes these updates to the frontal cortex—under the assumption that neuronal populations here encode working memory for the current state of the world (Goldman-Rakic et al., 1992). The functional anatomy in Figure 4 should not be taken too seriously—it is just used to illustrate the segregation and reciprocal message passing that follows from the computational logic of variational Bayes.

### Action selection

The second variational update is a softmax function of the expected value of competing choices under the current state. Figure 4 places this update in the striatum, where the expected value of a policy requires posterior beliefs about the current state from prefrontal cortex and expected precision from the ventral tegmental area. Crucially, this is exactly the softmax choice rule that predominates in QRE theory and other normative models (Haile et al., 2008). Again, it is remarkable that this rule follows directly from basic variational principles. However, utilitarian formulations overlook the symmetry between the expected value over states—that provides the value of a choice, and the expected value over choices—that provides the value of a state. In other words, there are two expected values, one for action *Q* · $\overset{⌢}{s}$ and one for perception *Q*^*T*^ · $\overset{⌢}{\pi }$. Furthermore, the expected value over choices *and* states $\overset{⌢}{\pi }$^*T*^ · Q · $\overset{⌢}{s}$_*t*_ specifies the optimal precision or inverse temperature, which is overlooked in classic treatments. Neurobiologically, the softmax policy updates would correspond to a winner-take-all or biased competition among competing choices or policies, where competition is modulated by precision. This is the second key role of precision; namely, to modulate the selection of competing representations of future action: cf. (Cisek, 2007; Frank et al., 2007; Jocham et al., 2012).

### Evaluating precision

The final variational step estimates the precision of prior beliefs about policies, using posterior expectations about hidden states and choices. We have associated expected precision with dopaminergic projections from the ventral tegmental area (and substantia nigra), which must be in receipt of messages from the prefrontal cortex and striatum. One of the key insights, afforded by the variational scheme, is that precision has to be optimized. So what would happen if (estimated) precision was too high or too low? If precision was zero, then perception would be unbiased and represent a veridical representation of worldly states. However, there would be a failure of action selection in the sense that the value of all choices would be the same. One might plausibly associate this with the pathophysiology of Parkinson's disease—that involves a loss of dopaminergic cells and a poverty of action selection. Conversely, if precision was too high, precise choices are made but there would be a predisposition to false perceptual inference—through the augmentation of optimism bias. This might be a metaphor for the positive symptoms of schizophrenia, putatively associated with hyper-dopaminergic states (Fletcher and Frith, 2009). In short, there is an optimal precision for any context and the expected precision has to be evaluated carefully on the basis of current beliefs about the state of the world.

**Figure 5:**
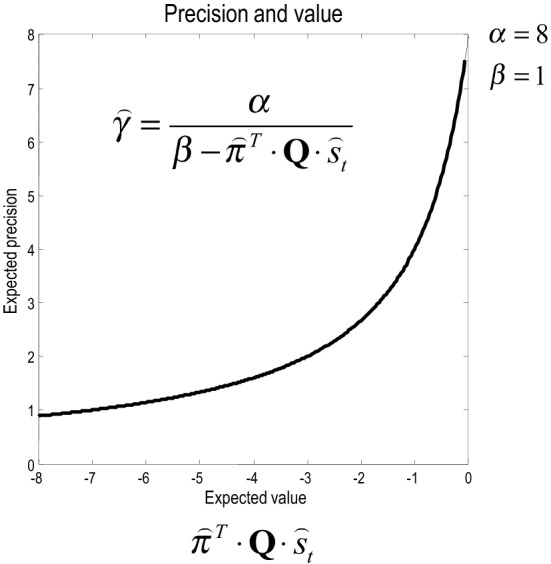
**The strictly increasing, monotonic relationship between expected precision and expected value**. Note that value never exceeds zero. This is because a Kullback–Leibler divergence can never be less than zero; by Gibbs inequality.

Inspection of the update for expected precision shows that it is an increasing asymptotic function of value, expected under current beliefs about states and choices (see Figure 5). This means that the optimal precision depends upon the attainability of goals: if a goal cannot be obtained from the current state, then precision will be small—reflecting a reduced confidence in predictions about behavior. Conversely, if there is a clear and precise path from the current state to a goal, then precision will be high. This means that precision reports the attainability of goals in terms of value. Mathematically, value can never be greater than zero (because the KL divergence is always non-negative). This means that precision increases to its upper bound of α, when value increases to zero (see Figure 5). In short, precision reports the expected value over states and policies and plays a dual role in biasing perceptual inference and action selection: on the one hand, it biases perceptual inference toward prior beliefs about future (choice dependent) outcomes. On the other hand, it encodes the confidence that a goal can be attained and increases the precision of action selection.

In summary, this section has considered the implications of variational Bayes for cognitive architectures and functional anatomy. The mean field assumption, enforced by the combinatorics and intractability of exact Bayesian inference, implies a segregation of inference into separable cognitive processes and their neuronal substrates (functional segregation). The particular mean field assumption used here implies distinct perceptual, choice and evaluation processes that can be associated with distributed cortical and subcortical systems in the brain. Crucially, every system (encoding the sufficient statistics of a marginal distribution) must receive signals from every system to which it sends signals. We will now look more closely at this reciprocal message passing.

## Decision-making under uncertainty

This section looks at simulated decision-making using the scheme above. The focus here will be on the circular dependencies between representations of hidden states and precision. This circular causality is one of the most important features of the variational scheme and means that one can consider not just the computational role of precision but also how it is controlled by the representations (posterior expectations) it optimizes.

Figure 2 (lower panels) provides an example of a simple “limited offer” game in which the agent has to choose between a low offer—that might be withdrawn at any time—and a high offer—that may replace the low offer with some fixed probability. The problem the agent has to solve is how long to wait. If it waits too long the low offer may be withdrawn and it will end up with nothing. Conversely, if it chooses too soon, it may miss the opportunity to accept a high offer. The probabilistic contingencies are shown in Figure 2 in terms of control dependent transition probabilities *B*(*u_t_*), where there are two control states (reject or accept) and five hidden states (low offer, high offer, no offer, accepted low offer, and accepted high offer). We can specify prior goals over the final states with a softmax function of utilities. Unless otherwise stated we will use:

(9) $$P(s_{T}|\theta )=c=\sigma \left({\left[1,1,1,2,4\right]}^{T}\right)$$

This means the agent believes it will accept the high offer exp (4 − 2) = 7.39 times more than the low offer, which, in turn is exp (2 − 1) = 2.718 times more likely than having accepted neither. To make things more interesting, we increased the probability of offer withdrawal with time such that the hazard rate: $r=1-{\left(1-\frac{1}{16}\right)}^{t}$. This also illustrates time-dependent transition probabilities that the variational scheme can handle with ease. Finally, the probability that a low offer changes into a high offer (provided it is not withdrawn) was fixed so that the probability of receiving a high offer over *T* = 16 trials was a half. This means the hazard rate in Figure 2 becomes $q=(1-r)\cdot (1-{\left(1-\frac{1}{2}\right)}^{1/T})$. For simplicity, we assumed the sensory mapping was the identity matrix such that *A* = *I*.

**Figure 6:**
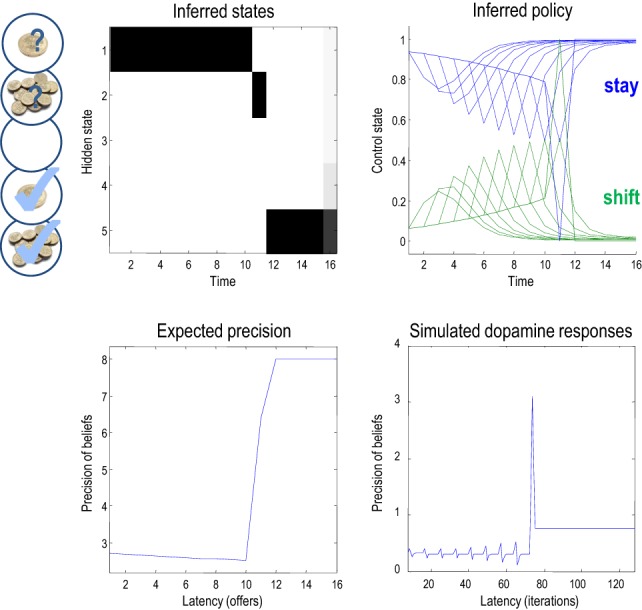
**This figure shows the results of a simulation of 16 trials, where a low offer was replaced by high offer on the 11th trial, which was accepted on the subsequent trial**. The **upper left** panel shows the expected states as a function of trials or time, where the states are defined in Figure 2. The **upper right** panel shows the corresponding expectations about control in the future, where the dotted lines are expectations during earlier trials and the full lines correspond to expectations during the final trial. Blue corresponds to reject (stay) and green to accept (shift). The lower panels show the time-dependent changes in expected precision, after convergence on each trial (**lower left**) and deconvolved updates after each iteration of the variational updates (**lower right**).

Figure 6, shows the results of a single game after iterating the variational updates of the previous section. In this example, the low offer was replaced with a high offer on the eleventh trial, which the agent accepted. It accepts because this is most probable choice—in the face of a high offer—under its prior beliefs that it is most likely to have accepted the higher offer at the end of the game. The expected probabilities of staying (rejecting) or shifting (accepting) are shown in the upper right panel (in green and blue, respectively), as a function of time for each trial (dotted lines) and the final beliefs (full lines). The interesting thing here is that prior to the high offer, the agent believes that it will accept the low offer three or four trials in the future. Furthermore, the propensity to accept (in the future) increases as time goes on (see dotted lines). This means that it waits, patiently, because it thinks it is more likely to accept an offer in the future than to accept the current offer.

The expected precision of these posterior beliefs is shown in the lower left panel and declines gently until the high offer is made. At this point the expected precision increases markedly, and then remains constant until the end of the game (at its maximum value of eight). This reflects the fact that the final outcome is assured with a high degree of confidence, once the high offer has been made and subsequently accepted. These precisions are the expected precisions after convergence of the variational iterations. The equivalent dynamics in the lower right panel show the expected precision over all updates in terms of simulated dopamine responses. These responses are a least squares deconvolution of the variational updates using an exponentially decaying kernel with a time constant of eight iterations. In other words, convolving the simulated dopamine responses with an exponential decay function would reproduce the Bayes optimal updates. This (de)convolution accounts for the postsynaptic effects of dopamine that, we imagine, decay exponentially after its release. The resulting updates are quite revealing and show phasic responses to the arrival of new sensory information that converge to tonic values, which minimize free energy.

**Figure 7:**
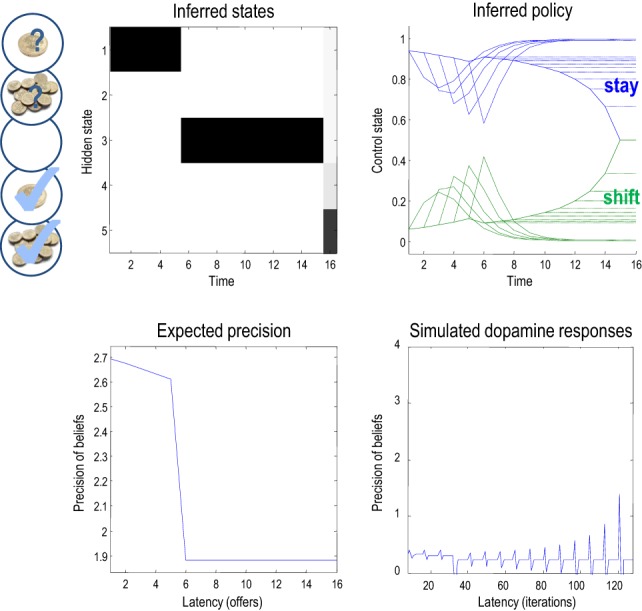
**This figure uses the same format as the previous figure; however, here, the low offer was withdrawn on the fifth trial, leading to a decrease in expected precision**. Note the difference (divergence) between the expected states on the 15th (penultimate) and 16 (final) trial. It is this large divergence (or more exactly the divergence between distributions over the final state) that leads to a small value and associated precision.

This pattern of precision encoding can be compared with another realization, in which the low offer was withdrawn after the fourth trial: Figure 7 shows the results of this simulation, where the expected control states and precision are exactly the same as in the previous simulation, until the offer is withdrawn. At this point, the agent moves to the no-offer state and remains there until the end of the game. Notice that there is still an increasing propensity to accept, even though the agent knows that accepting is futile. This is because all allowable policies entail a choice but with no preference for when that choice is made. This is because neither the entropy nor the expected utility of the final state is affected by subsequent choices. In this instance, precision falls at the point the offer is withdrawn and remains low until the last trial. Interestingly, at the point the offer is withdrawn, there is a profound suppression of simulated dopamine firing, followed by phasic bursts on subsequent cues that gently increase with the increasing probability of choosing—despite the fact that nothing can be changed. This illustrates the interdependency of expectations about precision and hidden states of the world—which change after the offer has been withdrawn. Many readers will have noticed a similarity between the dynamics of precision and the firing of dopaminergic cells in reinforcement learning paradigms, which we discuss further in (Friston et al., under review).

For people familiar with previous discussions of dopamine in the context of active inference, the correspondence between precision and dopaminergic neurotransmission will come as no surprise—exactly the same conclusions have been reached when examining predictive coding schemes (Friston et al., 2012b) and hierarchical inference using volatility models (Mathys et al., 2011). “In brief, the emergent role of dopamine is to report the precision or salience of perceptual cues that portend a predictable sequence of sensorimotor events. In this sense, it mediates the affordance of cues that elicit motor behavior (Cisek, 2007); in much the same way that attention mediates the salience of cues in the perceptual domain.” (Friston et al., 2012b, p. 2).

## Temporal discounting and marginal utility

**Figure 8:**
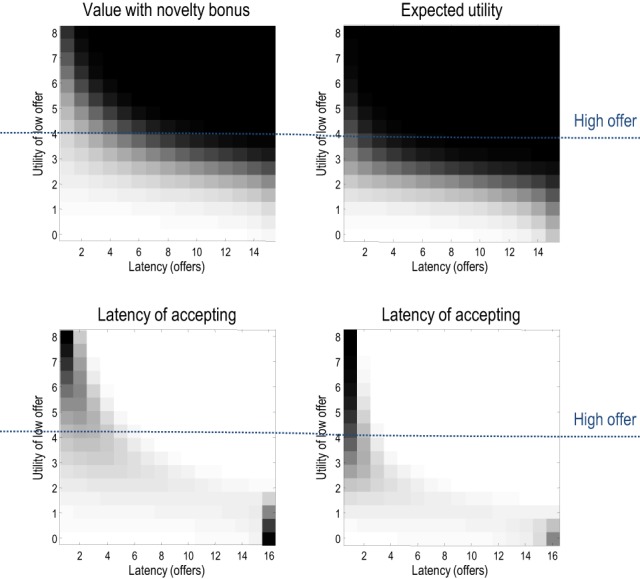
**The upper panels show the probability of accepting with (left) and without (right) the entropy or novelty part of value, where the low offer remained available and action was precluded**. These probabilities are shown as a function of trial number and the relative utility of the low offer (white corresponds to high probabilities). The lower panels show the same results but in terms of the probability distribution over the latency or time to choice. Note that including the entropy in value slightly delays the time to choice—to ensure a greater latitude of options. This is particularly noticeable in the ambiguous situation when the low offer has the same utility as the high offer (of four).

This section considers the relative contribution of entropy (exploration) and expected utility to choice behavior and how these contribution change with context and time. Generally, when relative utilities are large, they will dominate value (overshadowing entropy) and behavior will conform to expected utility theory. Figure 8 shows this numerically in terms of the probability of accepting over successive trials with, and without, the entropy term. Here, we precluded withdrawal of the low offer (and its acceptance) and increased the utility of the low offer from zero to eight. Inspection of the upper panels shows that the choice probabilities are essentially the same—with a tendency to wait until the last trial until the low offer becomes more attractive than the high offer (at a utility of four). However, there are subtle differences that are revealed in the lower panels.

These panels show the equivalent results but now in terms of the probability distribution over the latency or number of trials until an offer is accepted. This is simply the cumulative probability of waiting until a particular latency, times the probability of accepting at the latency in question. Here, one sees a slight increase in the latency when value includes the entropy term. This reflects the fact that accepting an offer precludes other outcomes and therefore reduces the entropy of the distribution over final states. Intuitively, there is value in keeping ones options open: cf. a novelty bonus (Krebs et al., 2009).

**Figure 9:**
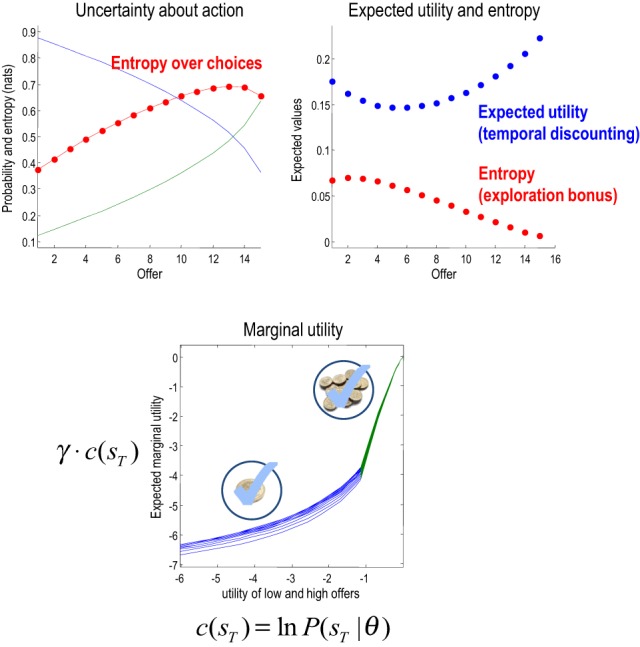
**Upper left panel:** the probability of accepting an offer as a function of time or trials. Note that the probability of accepting (green) increases over time to approach and surpass the probability of rejection. This produces an increase in the uncertainty about action—shown in red. **Upper right panel**: these are the expected utility and entropy components of expected value as a function of trial number. The key result here is the time-dependent change in expected utility, which corresponds to temporal discounting of the expected utility: i.e., the expected utility of the final state is greater when there are fewer intervening trials. **Lower panel**: the marginal utility of the high offer (green) and low offer (blue) as a function of the relative utility of the high offer. Marginal utility is defined here as expected utility times expected precision. The multiple curves correspond to the marginal utilities as a function of trial number (and do not differ greatly because expected precision changes more slowly over time—for a given utility—than it changes over utility—for a given time).

Figure 9 shows the underlying changes in entropy and expectations as a function of trial number (with a low offer utility of two). The upper left panel shows the probability of staying or accepting and the associated uncertainty or entropy of beliefs about the policy. One can see that this uncertainty increases as the propensity to accept increases. When the agent has in mind a 50–50 probability of accepting, the entropy peaks, shortly before the last offer. The entropy (red) and expected utility (blue) underlying these choices are shown in the right panel and demonstrate—in this example—a complementary dependency on time. As time progresses, the expected utility first falls and then increases, while the entropy does the converse. This suggests that the agent believes it is more likely to secure an offer later in the game, because it now knows the offer has not been withdrawn; in other words, the possibility of an early withdrawal cannot be discounted at the beginning of the game.

This dynamic speaks directly to temporal discounting in inter-temporal choice: consider the expected utility on the eighth trial. This is the utility of a final outcome eight trials in the future. Notice that this is substantially less than the expected utility of the final outcome two trials in the future. In other words, the expected utility of the outcome decreases, the further it recedes into the future. This is the essence of temporal discounting, which—in this example—can be explained simply by prior beliefs that the offer will be withdrawn before the final outcome is reached. This withdrawal probability is known as a *hazard function*, whose rate changes with time in our example (the parameter *r* in Figure 2).

### Temporal discounting

Temporal discounting is an emergent property of Bayes optimal inference about choice behavior that depends upon the generative model and, implicitly, prior beliefs about time sensitive contingencies—or at least it can be formulated as such (Sozou, 1998). The form of temporal discounting depends upon the generative model and can be quite complicated. This is because the discounting of expected utility depends upon inference about the current state, future choices and precision—all of which change with time in an interdependent fashion. Having said this (economic) hyperbolic discounting can be derived under a simple generative model of losing a reward, given exponential priors on the hazard rate (Sozou, 1998). Although hyperbolic (or exponential) discounting may be sufficient for descriptive purposes, simply optimizing a temporal discounting parameter (Daw and Touretzky, 2002), in light of observed behavior, cannot disambiguate among the prior beliefs an agent may entertain. To understand the nature of temporal discounting, one has to understand the generative model upon which that discounting is based—and use observed choice behaviors to select among competing models or hypotheses.

### Marginal utility and precision

We have been careful to distinguish between utility ln *P*(*s_T_*|θ) = *c*(*s_T_*)—an attribute of the final state and value *Q*(*ũ*|*s_t_*)—an attribute of choices available from the current state. This means that the value of the current state depends upon how easy it is to access the final state. Furthermore, the ensuing choice depends upon precision, suggesting that the effect of value on choice can be expressed in terms of an effective utility γ · *c*(*s_T_*) that we will call *marginal utility* (for consistency with economic theory). Assuming the entropy term in Equation (4) small enough to be ignored, it is easy to see that expected marginal utility directly informs choices:

(10) $$\ln P\left(\tilde{u}|s_{t}\right)=\sum_{s_{T}}\underset{\text{expected marginal utility}}{\underset{︸}{P(s_{T}|s_{t},\tilde{u})(\gamma \cdot c(s_{T}))}}$$

Generally, as the utility of a particular final state increases, precision increases more slowly—because the implicit distribution over final states is less likely to be realized. Intuitively, the marginal utility depends on the confidence that a goal can be reached. This leads to a convex relationship between marginal utility and utility: cf. the law of diminishing marginal utility (Kauder, 1953). The lower panel of Figure 9 illustrates this relationship. Here, we increased the relative utility of the high offer from two to eight and evaluated the marginal utility of accepting the low and high offers (by precluding offer withdrawal and action). The result is a characteristic convex relationship, in which marginal utility decreases more slowly with the utility of the high offer—reaching its maximum at zero. Conversely, the marginal utility of the low offer decreases more slowly as the utility of the low offer falls. In the current setup, this asymmetry results from the nature of utility and its dependency upon precision. However, there may be interesting connections here with Prospect Theory (Kahneman and Tversky, 1979) that appeal to a reference point for utility—defined here in terms of equiprobable outcomes.

In summary, many classic phenomena in utilitarian and economic theory resurface here as natural consequences of Bayes optimal (active) inference under a relatively simple generative model. This is potentially important, because choice behavior can, in principle, be used to adjudicate among alternative models used by subjects.

## Conclusion

This paper has considered agency from a rather elementary and formal perspective; namely, that a sense of agency rests upon prior beliefs about how one will behave. Irrespective of how these beliefs are described, they must—in some sense—entail the belief that our behavior will converge on outcomes that define who we are—in terms of our characteristic states. This can be formalized in terms of prior beliefs that controlled state transitions minimize a relative entropy or KL divergence—endowing behavior with a purpose that can be characterized by the states we believe should be occupied. The ensuing scheme appears to have construct validity in relation to normative accounts in psychology and economics. Furthermore, the computational anatomy afforded by variational Bayes fits comfortably with neuronal message passing in the brain.

In reinforcement learning, there is an important distinction between model-free and model-based systems (Daw et al., 2005). In contrast, active inference is quintessentially model-based—so does this preclude model-free schemes? Active inference accommodates the distinction between model-free and model-based by placing model-free schemes at the lower levels of hierarchical generative models. This enables higher levels to contextualize lower level (reflexive or habitual) inference and consequent action selection. We have not addressed this issue in this paper; largely because our focus has been on inference about hidden states, while learning corresponds to optimizing the parameters of the generative model—such as the probability transition matrices that encode environmental contingencies and which hidden states can and cannot be controlled.

The arguments in this paper are based upon—and lead to—a number of points, which we now briefly rehearse:

- Optimal behavior can be cast as a pure inference problem, in which valuable outcomes are defined in terms of prior beliefs about future states. However, exact Bayesian inference (perfect rationality) cannot be realized physically, which means that optimal behavior rests on approximate Bayesian inference (bounded rationality).
- Variational free energy provides a bound on Bayesian model evidence (marginal likelihood) that is optimized by bounded rational behavior. This requires (approximate Bayesian) inference on both hidden states of the world and (future) control states. This mandates beliefs about action (control) that are distinct from action *per se*—beliefs that entail a precision.
- These beliefs can be cast in terms of minimizing the relative entropy or divergence between prior goals—over final states—and conditional distributions, given the current state of the world and future choices.
- Value can be equated with negative divergence and comprises entropy (exploration or novelty bonus) and expected utility (utilitarian) terms that account for exploratory and exploitative behavior respectively.
- Beliefs about the state of the world depend upon expected value over choices, while beliefs about choices depend upon expected value over states. Beliefs about precision depend upon expected value under both states and choices.
- Precision has to be optimized to balance prior beliefs about choices and sensory evidence for hidden states. In other words, precision has to nuance an inherent optimism bias when inferring the current state of the world.
- Variational Bayes provides a formal account of how posterior expectations about hidden states of the world, control states and precision depend upon each other; and may provide a metaphor for message passing in the brain.
- Variational Bayes induces distinct probabilistic representations (functional segregation) of hidden states, control states and precision—and highlights the role of reciprocal message passing. This may be particularly important for expected precision that is required for optimal inference about hidden states (perception) and control states (action selection).

One might ask why these conclusions do not follow from normative accounts of optimal behavior. One reason is that normative accounts do not distinguish between action and beliefs about action (control). These beliefs entail both content (expectations) and uncertainty (precision). This means that both expectations about behavior and the precision of these beliefs have to be optimized. It is the optimization of precision that provides a complete account of bounded rationality (approximate Bayesian inference) and a putative account of the control of dopaminergic firing; cf. (Gurney et al., 2001).

This account considers dopamine to report the precision of divergence or prediction errors (in their nuanced or non-classical sense) and partly resolves the dialectic between dopamine as reporting reward prediction errors (Schultz et al., 1997) and the predictability of rewards (Fiorillo et al., 2003; Redgrave and Gurney, 2006; Schultz et al., 2008). The notion that dopamine encodes precision is now receiving support from several lines of evidence; from purely theoretical treatments of hierarchical Bayesian inference (Mathys et al., 2011), from theoretical neurobiology (Frank et al., 2007; Fletcher and Frith, 2009; Friston et al., 2012b; Pellicano and Burr, 2012) and from empirical studies (Fiorillo et al., 2008; Coull et al., 2011; Galea et al., 2012; Zokaei et al., 2012). Having said this, a proper validation of active inference will require careful model comparison using empirical choice behaviors and a detailed mapping between putative model variables and their neuronal correlates.

Indeed, the aim of this work was to provide a comprehensive but formal model of choice behavior that contextualizes decisions in the more general setting of embodied or active inference about states of the world; e.g., (Pezzulo and Castelfranchi, 2009). In this setting, the ability to compare different formulations of approximate Bayesian inference (in terms of different mean field approximations and prior beliefs) becomes crucial—because different formulations correspond to different hypotheses about how subjects optimize their behavior. We hope to use Bayesian model selection to characterize individual subjects, in terms of their prior beliefs using generative models of the sort introduced in this paper. This may be useful in the study of intersubject variability or indeed differences between normal subjects and those with psychiatric syndromes or addictive behaviors. The advantage of having a variational scheme with dynamics (that can be applied to these models) is that, in principle, one can localize the neuronal correlates of implicit Bayesian updates with neuroimaging. More generally, the theoretical approach adopted in this paper highlights the necessarily intimate relationship between inferring states of the world and optimal behavior (Toussaint and Storkey, 2006; Gläscher et al., 2010), the confidence or precision of that inference (De Martino et al., 2012), and the functional plurality of dopaminergic neuromodulation (Schultz, 2007).

In terms of leveraging active inference to further understand the neurobiology of decision-making, there are several predictions that could be explored—using either choice behavior or functional neuroimaging. One key prediction is that choices will systematically maximize the entropy over outcomes that have the same (relative) utility. In principle, it should be possible to design behavioral experiments that manipulate entropy and expected utility in an orthogonal fashion, to establish whether entropy represents an intrinsic drive. Furthermore, transcribing this sort of paradigm to fMRI should establish the validity of the putative functional segregation implied by the variational message passing scheme considered above. Indeed, we have used the game described in this paper as the basis of an fMRI experiment—and will be reporting the results in the near future. The neurobiological plausibility of variational message passing remains something of an open question. However, there is one comforting point of convergence between variational Bayes and more neurobiologically plausible predictive coding schemes (Bastos et al., 2012): this is the fact that the solution for both is exactly the same. In other words, it may be possible to formulate variational Bayes using neuronal dynamics that implement a gradient descent on variational free energy. Interestingly, this is precisely the (Variational Laplace) scheme used routinely in data analysis (Friston et al., 2007).

### Conflict of interest statement

The authors declare that the research was conducted in the absence of any commercial or financial relationships that could be construed as a potential conflict of interest.

## Footnotes

^1^In this paper, beliefs about states refer to a probability distribution over states.

## Appendix

The variational energies associated with each subset of hidden variables are derived by isolating terms in the generative model that depend upon the subset in question and evaluating their expectation, under their Markov blanket:

$$\begin{array}{l} V\left(s_{t}\right)=E_{Q\left(\psi \backslash s_{t}\right)}[\ln P\left(o_{t}|s_{t}\right)+\ln P\left(s_{t}|d\right)+\ln P\left(s_{t}|s_{t-1},a_{t-1}\right)+\ln P\left(\tilde{u}|s_{t},\gamma \right)]\\ \;=\ln A^{T}\cdot {\overset{⌢}{o}}_{t}+\left[t=0\right]\cdot \ln d+\left[t>0\right]\cdot \ln B\left(a_{t-1}\right)\cdot {\overset{⌢}{s}}_{t-1}+\overset{⌢}{\gamma }\cdot Q^{T}\cdot \overset{⌢}{\pi }\\ V\left(\tilde{u}\right)=E_{Q\left(\psi \backslash \tilde{u}\right)}\left[\ln P\left(\tilde{u}|s_{t},\gamma \right)\right]\\ \;=\overset{⌢}{\gamma }\cdot Q\cdot {\overset{⌢}{s}}_{t}\\ V\left(\gamma \right)=E_{Q\left(\psi \backslash \gamma \right)}\left[\ln P\left(\tilde{u}|s_{t},\gamma \right)+\ln P\left(\gamma |\beta \right)\right]\\ \;=\gamma \cdot {\overset{⌢}{\pi }}^{T}\cdot Q\cdot {\overset{⌢}{s}}_{t}+\left(\alpha -1\right)\ln\gamma -\beta \gamma \\ \;=\ln Q\left(\gamma \right)=\left(\alpha -1\right)\ln\left(\gamma \right)-\overset{⌢}{\beta }\gamma -\ln Z_{\gamma }\; \end{array}$$

The Iverson brackets [*t* = 0] return a value of one when the expression is true and zero otherwise.
